# Activity of Cefiderocol Alone and in Combination with Levofloxacin, Minocycline, Polymyxin B, or Trimethoprim-Sulfamethoxazole against Multidrug-Resistant Stenotrophomonas maltophilia

**DOI:** 10.1128/AAC.00559-20

**Published:** 2020-08-20

**Authors:** M. Biagi, A. Vialichka, M. Jurkovic, T. Wu, A. Shajee, M. Lee, S. Patel, R. E. Mendes, E. Wenzler

**Affiliations:** a College of Pharmacy, University of Illinois at Chicago, Chicago, Illinois, USA; b JMI Laboratories, North Liberty, Iowa, USA

**Keywords:** cefiderocol, *Stenotrophomonas maltophilia*, synergy, antimicrobial combinations

## Abstract

The production of an L1 metallo-β-lactamase and an L2 serine active-site β-lactamase precludes the use of β-lactams for the treatment of Stenotrophomonas maltophilia infections. Preclinical data suggest that cefiderocol is the first approved β-lactam with reliable activity against S. maltophilia, but data on strains resistant to current first-line agents are limited, and no studies have assessed cefiderocol-based combinations. The objective of this study was to evaluate and compare the *in vitro* activity of cefiderocol alone and in combination with levofloxacin, minocycline, polymyxin B, or trimethoprim-sulfamethoxazole (TMP-SMZ) against a collection of highly resistant clinical S. maltophilia isolates. For this purpose, the MICs of cefiderocol, ceftazidime, levofloxacin, minocycline, polymyxin B, and TMP-SMZ for 37 S. maltophilia isolates not susceptible to levofloxacin and/or TMP-SMZ were determined. Nine strains with various cefiderocol MICs were then tested in time-kill experiments with cefiderocol alone and in combination with comparators. The only agents for which susceptibility rates exceeded 40% were cefiderocol (100%) and minocycline (97.3%). Cefiderocol displayed the lowest MIC_50_ and MIC_90_ values (0.125 and 0.5 mg/liter, respectively). In time-kill experiments, synergy was observed when cefiderocol was combined with levofloxacin, minocycline, polymyxin B, or TMP-SMZ against 4/9 (44.4%), 6/9 (66.7%), 5/9 (55.5%), and 6/9 (66.7%) isolates, respectively. These data suggest that cefiderocol displays potent *in vitro* activity against S. maltophilia, including strains resistant to currently preferred agents. Future dynamic and *in vivo* studies of cefiderocol alone and in combination are warranted to further define cefiderocol’s synergistic capabilities and its place in therapy for S. maltophilia infections.

## Abstract (precis)

The production of an L1 metallo-β-lactamase and an L2 serine active-site β-lactamase precludes the use of β-lactams for the treatment of Stenotrophomonas maltophilia infections. Preclinical data suggest that cefiderocol is the first approved β-lactam with reliable activity against S. maltophilia, but data on strains resistant to current first-line agents are limited, and no studies have assessed cefiderocol-based combinations. The objective of this study was to evaluate and compare the *in vitro* activity of cefiderocol alone and in combination with levofloxacin, minocycline, polymyxin B, or trimethoprim-sulfamethoxazole (TMP-SMZ) against a collection of highly resistant clinical S. maltophilia isolates.

## INTRODUCTION

The development of novel antimicrobials has improved the efficacy and reduced the toxicity associated with treating some important multidrug-resistant (MDR) Gram-negative pathogens, such as carbapenem-resistant *Enterobacterales* (1–3) and Pseudomonas aeruginosa (4–6). However, although Stenotrophomonas maltophilia is the most prevalent carbapenem-resistant Gram-negative bloodstream pathogen in the United States and is associated with significant morbidity and mortality (7, 8), treatment strategies for this pathogen have not advanced in more than a decade (9). This is due in large part to the myriad resistance mechanisms possessed by S. maltophilia, including aminoglycoside-modifying enzymes, multidrug efflux pumps, and two intrinsic, inducible β-lactamase enzymes, the L1 metallo-β-lactamase and the L2 serine active-site β-lactamase (9). This broad array of resistance mechanisms has confined treatment to agents with increasing reports of resistance, high toxicity, and limited data with which to guide optimal dosing strategies (10–15).

Cefiderocol is a novel catechol-substituted siderophore cephalosporin with potent activity against MDR Gram-negative pathogens producing an array of β-lactamases, including both serine enzymes and metalloenzymes (16). Multiple studies including approximately 1,000 isolates have reported promising results on the *in vitro* activity of cefiderocol against S. maltophilia, consistently demonstrating MIC_90_ values from 0.12 to 0.5 mg/liter (17–21). Additionally, *in vivo* murine thigh and lung infection models confirm the potent efficacy of cefiderocol against S. maltophilia (22, 23). Unfortunately, these *in vivo* analyses included few levofloxacin- and/or trimethoprim-sulfamethoxazole (TMP-SMZ)-resistant isolates and no minocycline-resistant isolates, and they did not evaluate the activity of cefiderocol relative to that of clinically relevant comparators such as levofloxacin, minocycline, or TMP-SMZ. Additionally, the role of cefiderocol-based combination regimens has not been explored to assess the potential for *in vitro* synergy against this difficult-to-treat pathogen. As such, the objective of this study was to evaluate and compare the *in vitro* activity of cefiderocol alone and its activity in combination with levofloxacin, minocycline, polymyxin B, or TMP-SMZ against a global collection of highly resistant clinical S. maltophilia isolates.

## RESULTS

The MIC_50_, MIC_90_, and MIC range of each agent against all 37 isolates are summarized in Table 1. All isolates (100%) were susceptible to cefiderocol, and its MIC_50_ and MIC_90_ values were the lowest among those of all agents, at 0.125 and 0.5 mg/liter, respectively. Minocycline was the only other agent to which ≥40% of isolates were susceptible, at 97.3%. Based on CLSI interpretative criteria for P. aeruginosa, 28/37 (75.7%) isolates were intermediate to polymyxin B and 9/37 (24.3%) were resistant. Only 6/37 (16.2%), 13/37 (35.1%), and 14/37 (37.8%) isolates were susceptible to ceftazidime, levofloxacin, and TMP-SMZ, respectively.

**TABLE 1:** Activities of cefiderocol and comparator agents against 37 clinical Stenotrophomonas maltophilia isolates nonsusceptible to levofloxacin and/or trimethoprim-sulfamethoxazole

Agent	MIC (mg/liter)			Susceptibility^*a*^ (%)		
	50%	90%	Range	S	I	R
Cefiderocol	0.125	0.5	<0.03 to 1	100	0	0
Ceftazidime	64	>128	1 to >128	16.2	2.7	81.1
Levofloxacin	8	>16	0.25 to >16	35.1	13.5	51.4
Minocycline	2	4	0.125 to 8	97.3	2.7	0
Polymyxin B^*b*^	0.5	>8	0.03 to >8	0	75.7	24.3
TMP-SMZ^*c*^	8	>8	0.03 to >8	37.8	0	62.2

a S, susceptible; I, intermediate; R, resistant.

b Based on CLSI interpretive criteria for Pseudomonas aeruginosa.

c Values given reflect the MIC of the trimethoprim component only.

Table 2 displays the MIC values of cefiderocol and comparator agents against the nine S. maltophilia isolates selected for time-kill experiments. Cefiderocol MICs spanned nearly every doubling dilution, from 0.03 to 1 mg/liter, and there was an adequate distribution of resistant phenotypes across the other four comparators. Five (55.5%) isolates were susceptible to levofloxacin (MIC range, 1 to >16 mg/liter), 8 (88.9%) were susceptible to minocycline (MIC range, 0.125 to 8 mg/liter), 6 (66.7%) were intermediate to polymyxin B (MIC range, 0.125 to >8 mg/liter), and 3/9 (33.3%) were susceptible to TMP-SMZ (MIC ranges, 0.25 and 4.75 to >8 and 152 mg/liter for TMP and SMZ, respectively). No cross-resistance between cefiderocol and the comparator agents was observed, since none of the nine isolates were susceptible to all five agents, and the isolate that was least susceptible to cefiderocol (SM-7) was not resistant to any other agent, while the isolate that was most resistant to the four comparators (SM-9) demonstrated the lowest cefiderocol MIC (0.03 mg/liter).

**TABLE 2:** MICs of cefiderocol and comparator agents against nine S. maltophilia isolates included in time-kill experiments

Isolate	MIC (mg/liter)				
	Cefiderocol	Levofloxacin	Minocycline	Polymyxin B	TMP-SMZ^*a*^
SM-1	0.25	2	2	2	>8
SM-2	0.5	1	1	4	>8
SM-3	0.03	4	0.125	0.25	0.5
SM-4	0.125	8	0.5	0.125	0.25
SM-5	0.5	1	4	0.25	>8
SM-6	0.25	>16	2	>8	8
SM-7	1	4	2	0.25	0.5
SM-8	0.125	>16	8	2	8
SM-9	0.03	>16	4	>8	8

a Values reflect the MIC of the trimethoprim component only.

**FIG 1:**
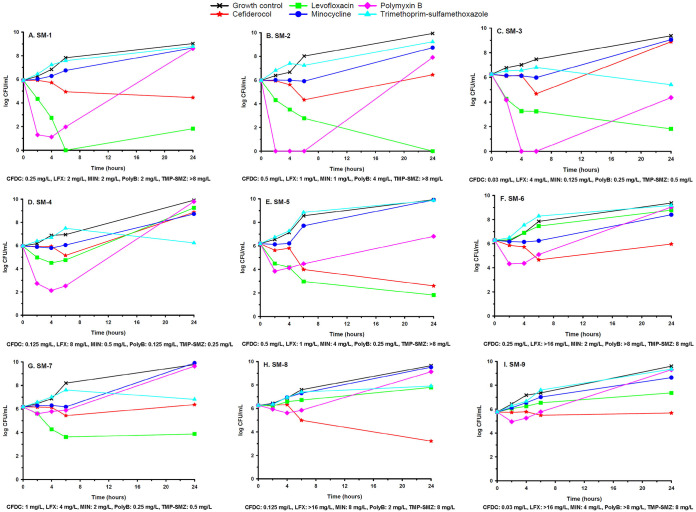
Mean bacterial concentration (expressed as log_10_ CFU per milliliter)-versus-time profiles for cefiderocol (4× MIC in all panels) and each comparator against nine S. maltophilia strains. Levofloxacin is shown at *fC*_max_ except in panels B and E (4× MIC). Minocycline is shown at *fC*_max_ except in panel C (4× MIC). Polymyxin B is shown at *fC*_max_ except in panels C, D, E, and G (4× MIC). TMP-SMZ is shown at *fC*_max_ except in panels C, D, and G (4× MIC). Curves represent average concentrations from triplicate experiments.

The results of monotherapy time-kill experiments with each agent alone at the highest concentration tested (4× MIC or the maximum concentration of the free, unbound fraction of the drug in serum [*fC*_max_]) are displayed in Fig. 1. Cefiderocol alone was bactericidal against 2/9 (22.2%) isolates (Fig. 1E and H). The mean (± standard deviation [SD]) decrease in the bacterial concentration from 0 to 24 h across all nine isolates exposed to cefiderocol at 4× MIC or *fC*_max_ was 0.05 ± 2.16 log_10_ CFU/ml. Levofloxacin alone was bactericidal against 4/9 (44%) isolates (Fig. 1A to C and E), and the mean (± SD) decrease from 0 to 24 h across all nine isolates was 1.36 ± 3.56 log_10_ CFU/ml. Minocycline, polymyxin B, and TMP-SMZ were not bactericidal against any isolate, regardless of the concentration tested.

**FIG 2:**
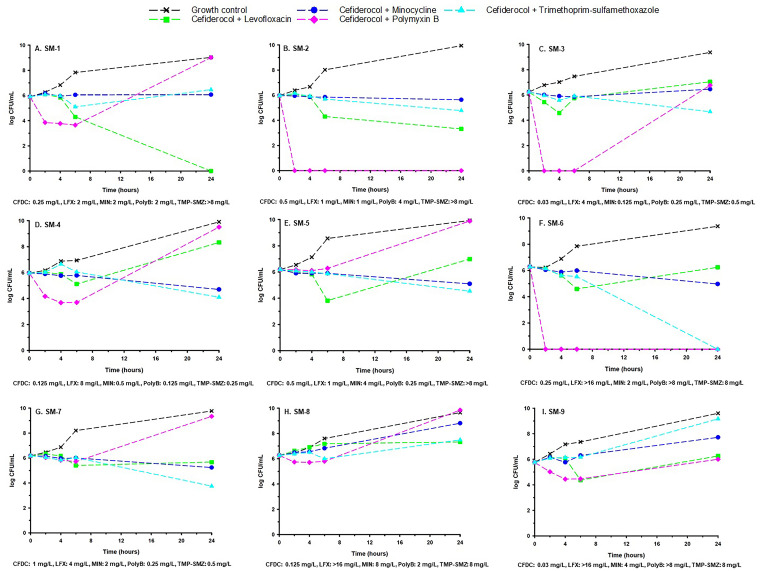
Mean bacterial concentration (expressed as log_10_ CFU per milliliter)-versus-time profiles for cefiderocol (½× MIC in all panels) in combination with each comparator against nine S. maltophilia strains. Levofloxacin is shown at either ¼× MIC (A to E and G) or *fC*_max_ (F, H, and I). Minocycline is shown at *fC*_max_ in all panels except C (4× MIC). Polymyxin B is shown at either *fC*_max_ (A, B, F, H, and I) or 4× MIC (C to E and G). TMP-SMZ is shown at *fC*_max_ in all panels except D (4× MIC). Curves represent average concentrations from triplicate experiments.

Based on results from individual time-kill experiments, a concentration of ½× MIC of cefiderocol was combined with ¼× MIC or *fC*_max_ of levofloxacin and either 4× MIC or *fC*_max_ of minocycline, polymyxin B, or TMP-SMZ (Fig. 2). The combination of cefiderocol plus levofloxacin was synergistic and bactericidal against 4/9 (44.4%) and 1/9 (11.1%) isolates, respectively (Fig. 2A, B, F, and G). The mean (± SD) decrease in the bacterial concentration after exposure to the combination from 0 to 24 h across all nine isolates was 0.39 ± 2.47 log_10_ CFU/ml. Synergy was observed in 2/3 (66.7%) levofloxacin-susceptible isolates and 2/6 (33.3%) levofloxacin-intermediate or -resistant isolates. The cefiderocol-plus-minocycline combination was synergistic against 6/9 (66.7%) isolates but was not bactericidal against any isolate (Fig. 2B to G). The mean (± SD) decrease after exposure to the combination from 0 to 24 h across all nine isolates was 0.0 ± 1.41 log_10_ CFU/ml. Cefiderocol combined with polymyxin B was synergistic and bactericidal against 5/9 (55.5%) and 2/9 (22.2%) isolates, respectively (Fig. 2B, C, E, F, and I), although the mean (± SD) bacterial concentration increased 0.67 ± 4.09 log_10_ CFU/ml from 0 to 24 h. Finally, cefiderocol combined with TMP-SMZ was synergistic and bactericidal against 6/9 (66.7%) and 1/9 (11.1%) isolates, respectively (Fig. 2B to G). Synergy was observed in 3/3 (100%) and 3/6 (50%) isolates susceptible or resistant to TMP-SMZ, respectively, and the mean (± SD) decrease from 0 to 24 h was 1.09 ± 2.70 log_10_ CFU/ml.

## DISCUSSION

The prevalence of serious infections due to S. maltophilia continues to increase concomitantly with its almost inescapable resistance, while the number of viable treatment options with reliable activity and acceptable safety profiles continues to decline. Cefiderocol is the first and only approved β-lactam agent to demonstrate reliable *in vitro* activity against Gram-negative pathogens expressing serine β-lactamase and metallo-β-lactamase enzymes. As such, there is a growing interest in the potential use of cefiderocol against S. maltophilia infections, although thorough evaluation of its activity against resistant isolates alone and in combination with other agents is crucial to establishing its role in this arena.

In the present study, the activity of cefiderocol was assessed alone and in combination against a unique panel of S. maltophilia isolates resistant to one or more currently preferred first-line treatment options. Susceptibility testing demonstrated that cefiderocol was highly potent against MDR S. maltophilia. Despite widespread resistance to other agents included in this study, the maximum cefiderocol MIC observed was 1 mg/liter, 2 log_2_ dilutions below the CLSI provisional susceptibility breakpoint of 4 mg/liter (24). Notwithstanding the fact that our sample was intentionally enriched with isolates resistant to levofloxacin and/or TMP-SMZ, these results are consistent with those of previous studies evaluating the *in vitro* susceptibility of S. maltophilia to cefiderocol (25–28).

This is the first study to directly compare the antibacterial activity of cefiderocol to those of currently preferred treatment options for S. maltophilia using time-kill experiments. Bactericidal activity was rarely observed in either monotherapy or combination time-kill experiments regardless of the agent(s) or concentration(s) tested, and strain-to-strain variability was visible across the nine isolates included. This is likely due to the slow-growing nature of S. maltophilia, the inherently static nature of time-kill experiments, and the drug concentrations utilized. Although supratherapeutic concentrations of cefiderocol as high as 4× MIC were utilized, these concentrations are still ≥10-fold lower than the *fC*_max_ values observed after a 2-g dose administered to healthy volunteers over 3 h (∼45 mg/liter) (29). Since the primary objective of this study was to evaluate synergy in combination with cefiderocol, drugs were utilized at concentrations multiplicative of the MIC for the respective isolate rather than at human physiologic concentrations. This approach allows for the evaluation of true synergy while maintaining a constant concentration-to-MIC ratio across pathogens (30), although it may underestimate the killing capacity possible at concentrations achievable in serum. Regardless, the inability of monotherapy to achieve bactericidal activity against S. maltophilia *in vitro* is consistent with the previous literature (31–33) and further supports the need to evaluate combination regimens against this difficult-to-treat pathogen.

Cefiderocol-based combinations were tested in 36 separate time-kill experiments (4 per isolate), and cefiderocol acted synergistically with another agent in 21/36 (58.3%) experiments but was bactericidal in just 4/36 (11.1%) combination experiments. In a majority of time-kill experiments, synergy was observed when cefiderocol was combined with either minocycline (66.7%), TMP-SMZ (66.7%), or polymyxin B (55.5%). Cefiderocol plus levofloxacin was the only combination for which synergy was not observed in at least 50% of experiments (44.4%). Further, although interstrain variability was high, cefiderocol in combination with TMP-SMZ achieved the largest average decrease in bacterial concentrations over the 24-h experiments, at 1.09 log_10_ CFU/ml, followed by polymyxin B at 0.67 log_10_ CFU/ml, levofloxacin at 0.39 log_10_ CFU/ml, and minocycline at 0.0 log_10_ CFU/ml. Although there appeared to be some correlation between susceptibility to the agent used in combination with cefiderocol and the achievement of synergy, the factors predictive of synergism with cefiderocol require further study. Additionally, the spectrum of synergy observed in this study warrants further investigation of these combinations in dynamic pharmacokinetic (PK)/pharmacodynamic (PD) models that can mimic humanized PK, elucidate dose-exposure-response relationships, and discover dosing regimens and/or combinations capable of achieving bactericidal activity against this elusive pathogen.

The strengths of our study include the use of a global collection of clinical isolates with resistance to levofloxacin and/or TMP-SMZ and the evaluation of cefiderocol both alone and in combination with currently preferred agents. Since we intentionally enriched our panel with resistant isolates, the rates of susceptibility to levofloxacin and TMP-SMZ in this study are not reflective of those encountered in routine clinical practice. Additional limitations of this study include the inherently static nature of 24-h time-kill experiments and the use of cefiderocol concentrations well below those that are clinically achievable.

In summary, cefiderocol displays potent *in vitro* activity against S. maltophilia, including strains resistant to current first-line agents. In time-kill experiments, minocycline, polymyxin B, and TMP-SMZ acted synergistically with cefiderocol against a majority of isolates. These results support the further investigation of cefiderocol both alone and in combination with these agents against S. maltophilia in more-complex *in vitro* and *in vivo* models in order to further define its place in therapy for this pathogen.

## MATERIALS AND METHODS

### Bacteria and susceptibility testing.

A panel of 37 clinical S. maltophilia isolates not susceptible to levofloxacin and/or TMP-SMZ collected through the SENTRY Antimicrobial Surveillance Program from 2017 to 2018 was included in all experiments (34). Species identification was confirmed at JMI Laboratories (North Liberty, IA) by standard biochemical tests and via matrix-assisted laser desorption ionization–time of flight mass spectrometry (MALDI-TOF MS) (Bruker Daltonics, Billerica, MA). Isolates included community- and nosocomially acquired strains collected from patients with various disease states across multiple continents (35). All isolates were maintained at –80°C in cation-adjusted Mueller-Hinton broth (CAMHB) (Teknova, Hollister, CA) with 20% glycerol and were subcultured twice on tryptic soy agar plates with 5% sheep blood prior to use.

Analytical-grade ceftazidime, levofloxacin, minocycline, polymyxin B, sulfamethoxazole, and trimethoprim powders were obtained commercially (Sigma-Aldrich, St. Louis, MO), and analytical-grade cefiderocol powder was provided by the manufacturer (Shionogi & Co., Ltd.). Stock solutions of each agent were freshly prepared as single-use aliquots at the beginning of each week and were kept frozen at –80°C. MICs were determined in triplicate via reference broth microdilution according to Clinical and Laboratory Standards Institute (CLSI) guidelines using the same 0.5 McFarland standard suspension (36). Cefiderocol MICs were determined using iron-depleted CAMHB (ID-CAMHB) as recommended elsewhere (24, 37) in custom-prepared MIC panels (International Health Management Associates, Schaumburg, IL). Modal MIC values are reported as MIC_50_, MIC_90_, and MIC range. Escherichia coli ATCC 25922 and Pseudomonas aeruginosa ATCC 27853 were used as quality control organisms. Susceptibility interpretations were based on 2020 CLSI interpretative criteria (document M100-S30) for activity against S. maltophilia for all agents except polymyxin B, for which results were interpreted on the basis of CLSI interpretative criteria for P. aeruginosa (24). Susceptibility breakpoints were as follows: for cefiderocol, ≤4 mg/liter; for ceftazidime, ≤8 mg/liter; for levofloxacin, ≤2 mg/liter; for minocycline, ≤4 mg/liter; and for TMP-SMZ, ≤2 and 38 mg/liter, respectively. A polymyxin B MIC of ≤2 mg/liter was considered intermediate given the lack of a susceptible category in CLSI document M100-S30.

### Time-kill experiments.

Time-kill experiments were performed in triplicate on the same day against a subset of nine S. maltophilia isolates selected to provide a range of cefiderocol MICs and a variety of phenotypic susceptibilities across comparator agents. Experiments were performed according to CLSI guidelines (38) modified using a final volume of 2 ml in deep-well, non-tissue-treated plates. A starting inoculum of ∼10^6^ CFU/ml was prepared by suspending 3 to 4 isolated colonies selected from a pure overnight culture in 5 ml of sterile saline and adjusting to a 0.5 McFarland standard; the suspension was subsequently incubated with agitation to ensure log-phase growth and was then diluted 1:100 in CAMHB. Colony counts were performed to ensure final inoculum densities. Time-kill experiments were performed stepwise as follows: cefiderocol, levofloxacin, minocycline, polymyxin B, and TMP-SMZ were tested alone at ¼, ½, 1, 2, and 4× MIC, unless any of these concentrations exceeded the respective drug’s *fC*_max_ value, in which case the *fC*_max_ was used. Additionally, if the MIC value was below the limit of quantitation (e.g., <0.03 mg/liter), then the lowest observed value was used (0.03 mg/liter). The *fC*_max_ values utilized simulated single doses of 750 mg levofloxacin (6.5 mg/liter) (39), 200 mg minocycline given intravenously (1 mg/liter) (40), 1.5 mg polymyxin B/kg of body weight (2.5 mg/liter) (41), and 400 and 2,000 mg TMP-SMZ, respectively, given intravenously (5 and 35 mg/liter) (42). The *fC*_max_ of TMP-SMZ simulated a 5-mg/kg dose of TMP administered to an 80-kg patient (42). Next, cefiderocol was tested at ½× MIC in combination with each comparator agent using the highest concentration of each individual agent from step 1 that displayed no meaningful activity compared to the drug-free control strain (≤1-log_10_ CFU/ml decrease from the starting inoculum at 24 h). A growth control without any antibiotic was included with each experiment. All cefiderocol-based experiments were performed using ID-CAMHB, including combination experiments, after an initial evaluation via MICs and time-kill analyses to ensure that the use of ID-CAMHB did not affect the activity of any comparator agent (data not shown). At the prespecified time points of 0, 2, 4, 6, and 24 h, aliquots of 20 μl were removed from the suspensions and serially diluted in log_10_ dilutions. A 50-μl aliquot was then plated onto MH agar plates using an automated spiral plater (Don Whitley WASP Touch; Microbiology International, Frederick, MD) and was incubated at 35°C for at least 24 h prior to enumeration. Colony counts were performed using an automated colony counter (ProtoCOL 3 Plus; Synbiosis, Frederick, MD). The theoretical lower limit of quantitation was 100 CFU/ml. Time-kill curves were generated by plotting the average bacterial concentration (expressed as log_10_ CFU per milliliter) against time to compare the 24-h killing effects of single agents alone and in combination. Bactericidal activity was defined as a ≥3-log_10_ CFU/ml reduction at 24 h from the starting inoculum, and synergy was defined as a ≥2-log_10_ CFU/ml difference between the combination and the most active single agent alone (38).
